# A Review of Dr. Senn’s Views on Elastic Constriction

**Published:** 1893-07

**Authors:** W. H. Elliott

**Affiliations:** Savannah, Ga.


### Southern Medical Record.

A MONTHLY JOURNAL OF MEDICINE AND SURGERY.

VOL. XXIII. ATLANTA, GA., JULY, 1893. No. 6. 7

### Original Articles.

### A REVIEW OF DR. SENN'S VIEWS ON ELASTIC CONSTRICTION.

BY W. H. ELLIOTT, M. D., SAVANNAH, GA.

Dr. Elliott addressed the Association as follows :

Mr. President—At the last meeting of the National Association of Railway Surgeons, I had the pleasure of hearing Dr. Senn deliver an experimental lecture on the subject of "Elastic Constriction as a Hæmostatic Measure." I was so impressed with the soundness of Dr. Senn's views, and the value of his suggestions, that I have decided to ask your attention to a review of his remarks.

I have not written out what I have to say on this subject, for the simple reason that Dr. Senn had not written out his lecture, and if I attempted to put my remarks in writing I could not do Dr. Senn justice. I will endeavor however, to do him as much justice as I can without having my remarks written.

You are all familiar with Esmarch's method, which consists in putting on an elastic bandage slowly and gradually, from the periphery up to a point just above where you are going to operate, so as to empty the limb of blood. He then puts on a

* Read before the Medical Association of Georgia, April 1893.

tube, or a narrow elastic band at that point, so as to cut off the circulation from the limb entirely, then removing the compression bandage, proceeds with the operation. He has fitly called this "The Bloodless Operation." Esmarch was not the inventor of the bloodless operation, but we are indebted to his genius for improving its technique, and for making this an established method, and popular procedure in surgery.

Dr. Senn maintains however, that there are two grave objections to the method as used by Esmarch. The first one of these is, that by forcibly compressing the blood out of the tissues by an elastic bandage, we are liable to send into the circulation or surrounding tissues pathogenic microbes. For instance, cancer cells, or the cells of sarcoma may thus be scattered and disseminated through the system, and also the cells of pus or of tuberculosis may be sent abroad to do damage elsewhere.

Dr. Senn's second objection is that constriction of the limb with a tube or narrow bandage at one point is liable to do injury first to the muscles, and secondly, to the nerves. He cited two instances, in one of which a paralysis lasting for three months, had resulted from linear constriction; and another instance where the paralysis had become permanent; and if the nerve is cut the functions of the muscles will be seriously interfered with.

I show you here a tube which is about the size, as far as I can judge from the picture, that Esmarch was in the habit of using in his method, and you are all familiar with the ordinary elastic bandage. I show you the Esmarch apparatus complete, except that the tube should have a chain on the end for locking. This latter is an unimportant matter because you can do it with a little piece of tape.

Now to obviate the objections to Esmarch's method, Dr. Senn offers two suggestions. One is not to empty the limb of blood by forcible compression, but by gravitation only. Simply take the arm or the leg that you propose to operate on, and elevate it from the operating table, keeping it up from one to two or more minutes, and you will be astonished how quickly the limb will be emptied, so that you will not loose much blood in the operation. Then he puts on the constrictor, which should not be a narrow one like Esmarch's tube. He uses an India rubber bandage, which should be at least two inches broad.

There are some points in regard to the manner in which the bandage should be applied. In the first place, constriction on the vessels must be applied *quickly*, so as to at once cut off the circulation in both arteries and veins. The reason for this is obvious. If you apply compression gradually you will stop circulatiou in the veins before you do in the arteries, and the limb is filling up with blood, which you will loose as soon as you cut. The constriction must therefore, be put on quickly, and *first on the side of the limb on which the blood vessels lie.* In the case of the arm you wlll make compression on the inner side. You pass the bandage on the inside of the arm, give it one good pull in order to make constriction rapidly and stop the circulation in both arteries and veins at once. Where the elastic constriction is put on the upper extremity, the most favorable position is just at the insertion of the deltoid, and if it cannot be placed there, then a little higher up. So too when operating on the lower extremity, the constriction should be applied on the middle of the thigh or above that point, and not near the knee joint. It was in a case where Dr. Senn applied the constrictor just above the knee joint that he had paralysis of the saphenous nerve resulting from it.

Another consideration in the application of the constrictor is, that you should not put it on near to any joint except perhaps the shoulder or hip. If you do, you run the risk of having paralysis after the operation. If you are obliged to put the bandage where the nerve is superficial, you can protect it from injury to some extent by placing a piece of gauze over it. This is Dr. Senn's suggestion. Dr. Marks of Milwaukee, says the gauze ought always be used in elastic constriction. The matter of applying it over the nerves is an important one, because linear constriction, as you know, with a narrow tube, is almost equivalent to nerve section, and the integrity of the nerves afterwards may not be restored.

While discussing this subject and studying Dr. Senn's views, it is only just to Esmarch that I should read an extract from his book; edition of 1887. He says: "Parts which contain unhealthy pus must not be firmly bandaged, for infecting matter may thereby be driven upwards into the cellular tissue and into the lymphatics. In such cases one must be satisfied with raising the limb on high for a few minutes before applying the bandage, so as to diminish the amount of blood in the vessels." These remarks are from Lister, but Esmarch quotes and endorses them. Although Esmarch in his book draws a picture of a tube and recommends it, he also resorts to broad constriction made by a few turns of a rubber bandage, and also by broad belt of Nicaise, both methods being shown in his illustrations.

Justice to Esmarch, requires that these facts should be stated, because they are in his book published in 1887. But at the same time, Dr. Senn could have no stronger endorsement of the position he takes than the facts in Esmarch's book. Within the last two or three weeks the correspondence between Dr. Senn and Esmarch has been published in the surgical department of the Railway Age, which settles the question at issue between the two gentlemen, and shows that the points of Dr. Senn are well founded.

Having decided upon the best method of rendering the limb bloodless, there are two practical questions to be considered. The first is: How long it is safe to continue elastic constriction if you do it the right way? Dr. Senn performed a series of experiments upon dogs where he applied constriction with a tube for a period varying from two honrs and twenty-five minutes to twenty-six hours. The operations were followed —especially those in the longer period—by more or less damage to the muscles and nerves, the animals being unable in many instances to use their legs for several days afterwards, and in one case where constriction was tightly applied for seventeen hours gangrene resulted. The practical deduction that Dr. Senn draws from his experiments is this: That the limit of safe constriction is two hours and a half, and he advises that that time should never be exceeded.

In the so-called bloodless operation, you all know that it is liable to be followed by free oozing, and in order to reduce this to a minimum, Dr. Senn advises that, for five minutes before the elastic constrictor is taken off, the limb should be again elevated ; that the wound, if it admits of it, should have its cavity filled with antiseptic gauze, and the flaps brought over and pressure made on them till the constrictor is removed. If in spite of this there is much oozing he recommends a douche as hot as can be borne.

In closing, I desire to append the following conclusions formulated by Dr. Senn in regard to "Elastic Constriction as a Hæmostatic Agent."

1. The use of the elastic bandage to secure a bloodless condition of a limb should be discarded, as compression of the parts affected may produce mechanically, dissemination of malignant tumors and microbic diseases.

2. A bloodless condition should be secured by elevation of the limb prior to constriction.

3. Constriction should be made with sufficient force to interrupt at once both the arterial and venous circulation.

4. Prevent venous stasis by constricting quickly, beginning pressure on the side of the limb supplied with the principal blood-vessels.

5. Linear or too firm constriction should be avoided, as they are liable to give rise to muscular injury and temporary or permanent paralysis due to harmful compression of a large nerve-trunk.

6. Elastic constriction of a limb for hæmostatic purposes should be diffused over an annular space not less than two inches in width, and can be made with least danger of injuring important structures by an elastic band for this purpose or an ordinary elastic bandage.

7. Circular constriction of a limb should be made, if possible, at a point where the long nerve-trunks are well protected by overlying muscles, and if this cannot be done on account of the site of operation, a thick compress of gauze should be interposed between the constrictor and the limb.

8. The vitality of the tissues when excluded from the circulation is endangered by prolonging the ischæmic condition for three or four hours, and gangrene may take place if constriction is continued for a long time.

9. The process of karyokinesis in tissues temporarily deprived of circulation by elastic constriction is unfavorably affected if constriction is continued for more than two hours.

